# Authors' reply: Meta‐analysis of oral antibiotics, in combination with preoperative intravenous antibiotics and mechanical bowel preparation the day before surgery, compared with intravenous antibiotics and mechanical bowel preparation alone to reduce surgical‐site infections in elective colorectal surgery (*BJS Open* 2018; 2: 185–194)

**DOI:** 10.1002/bjs5.50197

**Published:** 2019-07-23

**Authors:** S. T. McSorley, C. W. Steele, A. J. McMahon

**Affiliations:** ^1^ Academic Unit of Surgery, University of Glasgow, Glasgow Royal Infirmary, Alexandra Parade Glasgow G31 2ER UK

We thank our colleagues for their interest in our published meta‐analysis examining the impact of oral antibiotic bowel preparation on surgical‐site infection (SSI) following elective colorectal surgery^1^, and for pointing out an important issue: the possibility of including duplicate patients/cohorts when pooling observational data. As the authors state, the inclusion of duplicate data in pooled analyses will likely lead to false reinforcement of the reported treatment effect direction and size, and is therefore to be avoided^2^.

This issue is particularly relevant to the evidence surrounding the use of oral antibiotics and mechanical bowel preparation in colorectal surgery, as a large proportion of the more recent data comes from NSQIP and VASQIP data sets^3^, ^4^, ^5^, ^6^, ^7^, ^8^. The primary reporting of multiple cohort studies from such databases with overlapping time periods leads to great difficulty in determining which are likely to contain duplicate patients and data, and which combination of included studies leads to the least possible bias. This is illustrated by the fact that each of three recent meta‐analyses on the topic, including our own^1^, ^9^, ^10^, includes a different combination of cohort studies originating from NSQIP. Furthermore, from certain cohort studies included in all three, each meta‐analysis has utilized different subgroups, dependent on the outcome in question. This is even after study selection and agreement between multiple authors in each of the publications.

It was for this reason that a sensitivity analysis looking at the primary outcome of SSI, containing only RCTs considered to have high‐quality methodology, was performed in our own study. Indeed, as Meyer and colleagues suggest in their letter, the results were comparable to that which included the NSQIP cohort studies. As many of the secondary reported outcomes including anastomotic leak, reoperation, readmission and mortality could only be derived from the included cohort studies, it is possible that inclusion bias may prejudice those results. The similarity of the primary outcomes in the cohort and RCTs following synthesis is somewhat reassuring, although we accept that there was variation in findings with regard to organ‐space SSI between the included RCTs and cohort studies. Finally, we might expect that improvement in outcomes such as SSI might also be associated with a reduction in other negative outcome measures, although, given the nature of these studies, no causal implications can be drawn.

The authors agree that the inclusion of large cohort studies reported from the NSQIP data set is problematic; however, each of the three recent meta‐analyses included such studies alongside RCTs, and each reported similar results in terms of the primary outcome, postoperative SSI following elective colorectal surgery.
